# Primary ciliary dyskinesia and humoral immunodeficiency - what is the missing link?

**DOI:** 10.1186/2045-7022-3-S2-P11

**Published:** 2013-07-16

**Authors:** Mieke Boon, Mark Jorissen, Kris De Boeck, Isabelle Meyts

**Affiliations:** 1 UZ Gasthuisberg Leuven, Pediatric Pulmonology, Leuven, Belgium; 2 UZ Gasthuisberg Leuven, Otorhinolaryngology, Leuven, Belgium; 3 UZ Gasthuisberg Leuven, Pediatric Immunology, Leuven, Belgium

## Background

Primary ciliary dyskinesia (PCD) is a rare disorder (prevalence 1/20000), caused by congenital dysmotility of the respiratory cilia. Humoral immunodeficiency (HID) often presents in a similar way with recurrent ear, nose and sinopulmonary infections, not seldom evolving to chronic lung disease. Although isolated IgG subclass deficiencies and IgA deficiency are common conditions, Common Variable Immunodeficiency (CVID) is more rare with a prevalence ranging from 1/10000 to 1/50000.

## Method

We examined the coincidence of PCD with HID in a large cohort of patients with PCD. The diagnosis of PCD was confirmed by functional and structural evaluation of the cilia, including evaluation after ciliogenesis in culture, excluding secondary ciliary dyskinesia.

## Results

We report the coincidence of PCD with HID in 8 patients (4.6%). Table 1 represents the patient characteristics. Skorpinski et al already reported on the surprising association of PCD and CVID in a single patient. We have no explanation for this remarkable finding, but hypotheses can be postulated. Hematopoietic cells lack primary cilia, but they do express certain intraflagellar transport proteins needed for the formation of the immune synaps. Dysfunction of one of these proteins might cause PCD as well as HID. Ciliary proteins might play a role in B-cell proliferation or immunoglobulin class-switch and normal ciliary function might be needed for a fully intact immune response to antigen presentation. PCD could cause cytokine dysfunction, which could disturb the immunoglobulin secretion indirectly. Immune dysfunction may be a feature of PCD and explain part of the symptom complex. PCD genes could be modifier genes for HID genes or vice versa. Of course, it can be pure coincidence that some patients present with both disorders and that no causal relation is present. The diagnosis of HID can be important since immunoglobulin substitution is an effective treatment and therefore checking antibody titers should be recommended.

**Table 1:** 

Patient	Age (y)	Clinical presentation	Immuno deficiency	Laboratory results (before start of replacement therapy)						Treatment HID
				IgG (g/l)	IgG2 (g/m)	IgG3 (g/l)	IgA (g/l)	IgM (g/l)	Pneumococcal antibodies (before-after vaccination) (U/ml)	

1*	13	SI, C, B, CR, E	CVID	7,54 (6,35-14,89)	0,5 (0,63-3,0)	0,13 (0,17-0,88)	0,29 (0,46-2,51)	0,23 (0,47-2,2)	Type 3: 33-114, type 4: 38-23, type 9N: 6-33	SCIG
2*	16	C, B, CR	CVID	8,55 (4,78-11,29)	0,38 (0,72-3,4)	0,27 (0,13-1,33)	0 (0,35-1,9)	0,41 (0,34-1,34)	Type 3: 26-57, type 4: 8-54, type 9N:7-66	SCIG
3	32	SI, C, B, CR, E	IgG2 and IgG3 deficiency	8,3 (7-16)	1,8 (2,42-7,0)	0,18 (0,22-1,76)	1,37 (0,7-4,0)	0,37 (0,4-2,3)		SCIG
4	43	C, B, CR	IgG3 deficiency	9,34 (7,51-15,6)	3,38 (1,50-6,40)	0,11 (0,20-1,10)				Intermittent IVIG
5	62	SI, C, B, CR	IgG2 deficiency	6,64 (7,51-15,6)	1,14 (1,5-6,4)	0,96 (0,2-1,1)	1,43 (0,82-4,53)	2,71 (0,46-3,04)		Intermittent IVIG
6	33	C, B, CR, E	IgG3 deficiency	14,0 (7,51-15,6)	4,06 (1,17-7,47)	0,29 (0,41-1,29)				No IG treatment
7#	10	SI, C, B, CR, E	IgA deficiency, SPAD	14,5 (5,3-13,06)	1,27 (0,98-4,8)	0,59 (0,15-1,49)	0,29 (0,6-2,7)	2,56 (0,43 - 2,7)	Type 3: 30-21, type 4: 9-7, type 9N: 8-6	No IG treatment
8#	14	C, B, CR, E	IgA deficiency	12,6 (5,76-12,65)			0,56 (0,81-2,32)	1,28 (0,3-1,59)		No IG treatment

Legend to the figure: B: bronchiectasis, C: chronic cough, CR: chronic rhinosinusitis, E: recurrent ear infections, Si: situs inversus, SPAD: specific polysaccharide antibody deficiency, SCIG: subcutaneous immunoglobulin, IVIG: intravenous immunoglobulin, * and #pair of sisters. Normal reference values are reported between brackets.

## Conclusion

Patients with PCD may be at increased risk for coincident HID and a underlying link between both disorders might be present.

